# 吉非替尼对肺癌细胞株HCC827和H358放射敏感性的影响及其机制研究

**DOI:** 10.3779/j.issn.1009-3419.2012.06.02

**Published:** 2012-06-20

**Authors:** 子夜 高, 亮 庄, 元 陈

**Affiliations:** 430030 武汉，华中科技大学同济医学院附属同济医院肿瘤中心 Cancer Center, Tongji Medical College, Huazhong University of Science and Technology, Wuhan 430030, China

**Keywords:** 表皮生长因子受体, 肺肿瘤, 辐射耐受性, 吉非替尼, Epidermal growth factor receptor, Lung neoplasms, Radiation tolerance, Gefitinib

## Abstract

**背景与目的:**

表皮生长因子受体（epidermal growth factor receptor, EGFR）是决定放疗效应的一个重要因素，其过表达或是下游通路的激活与包括非小细胞肺癌在内的肿瘤的放疗抵抗相关，因而阻断EGFR的信号通路可能会增强放疗敏感性。本研究旨在探讨小分子EGFR酪氨酸激酶抑制剂吉非替尼能否增加肺癌细胞株HCC827和H358的放疗敏感性及其可能的机制。

**方法:**

选取HCC827和H358这两个非小细胞肺癌细胞株，分为单纯X线组和X线+吉非替尼两组。单纯X线组采用单纯X线照射，X线+吉非替尼组经1 μmol/L吉非替尼作用24 h后再行X线照射。克隆形成实验比较两株细胞中不同分组细胞放射敏感性，免疫荧光激光共聚焦显微镜观察X线照射后各时间点细胞核中的磷酸化H2AX（γ-H2AX）及EGFR焦点在细胞中的定位情况，Western blot法检测放疗后胞质胞核蛋白中EGFR的表达。

**结果:**

克隆形成实验中，H358细胞实验组与对照组在各放疗剂量点的SF2值分别为0.355和0.433；HCC827细胞实验组与对照组在各放疗剂量点的SF2值分别为0.223和0.242，差别不明显。激光共聚焦显微镜观察照射4 Gy后各时间段实验组H358细胞核中g-H2AX斑点相比对照组要多，且持续时间更长。而对照组和实验组的HCC827细胞g-H2AX斑点在各时间段并无明显差异；激光共聚焦显微镜观察照射4 Gy后对照组H358的EGFR蛋白在1 h内入核，而经吉非替尼处理后EGFR蛋白几乎不入核；实验组及对照组HCC827细胞的EGFR表达位置均在细胞质中，胞核中很少或者没有，可以认为并无入核现象；Western blot结果显示，H358细胞在经4 Gy放射处理后有入核现象，而预先经吉非替尼处理后，EGFR蛋白几乎不在核内表达而仍位于细胞浆内。对于HCC827细胞，实验组及对照组的EGFR蛋白均在细胞质中表达，胞核中很少或没有，且两组并无明显差异。

**结论:**

吉非替尼可增加肺癌细胞株H358的放射敏感性，这可能与其阻止放疗后EGFR入核、影响放疗后双链断裂（double strand break, DSB）修复有关；而对HCC827细胞无影响，可能与其放疗后EGFR不入核相关。

## Abstract (en)

**Background and objective:**

The epidermal growth factor receptor (EGFR) is an important determinant of tumor response to ionizing radiation. Elevated levels of EGFR expression and activity are frequently correlated with the radiotherapy resistance of tumors, including that of non-small cell lung cancer (NSCLC). The molecular inhibition of EGFR signaling is a promising therapeutic strategy for enhancing the cytotoxic effects of radiotherapy. The aim of the present study is to determine whether gefitinib, a selective EGFR tyrosine kinase inhibitor, can radiosensitize the NSCLC H358 and HCC827 cell lines. The study also aims to elucidate the mechanism, by which this drug restores the radiosensitivity of NSCLC cells.

**Methods:**

The two cell lines were divided into two groups, the X-ray and gefitinib-interfering groups. The former was irradiated with X-rays only, and the latter was treated with 1 μmol/L gefitinib for 24 h before irradiation under the same conditions as those for the first group. Clonogenic cell survival assay was performed to determine the radiosensitivity of both cell groups. Immunostaining for confocal microscopy was performed to observe nuclear γ-H2AX repair and EGFR foci after irradiation. Nuclear EGFR expression was also detected by Western blot analysis after radiotherapy.

**Results:**

The clonogenic cell survival assay revealed that the surviving fraction at 2 Gy (SF2) of the gefitinib-interfering group (0.355) was lower than that of the X-ray group (0.433) in H358 cells. There was no significant difference between the SF2 values of the two respective groups in HCC827 cells (0.223 *vs* 0.242). The confocal microscopy results found that gefitinib increased the average number of γ-H2AX foci after irradiation in H358 cells, but did not affect the foci in HCC827 cells. Based on confocal microscopy and Western blot analyses, gefitinib also inhibited the translocation of EFGR into the nuclei of H358 cells. However, EGFR was not observed in the nuclei of HCC827 cells for both treatment groups.

**Conclusion:**

Gefitinib enhances the radioresponse of H358 cells, which may be attributed to the suppression of EGFR transport into the nucleus. However, gefitinib does not affect HCC827 cells, where EGFR remains in the cytoplasm after irradiation.

肺癌是目前发病率和死亡率最高的恶性肿瘤，即使积极治疗，其总的5年生存率也仅有15%^[1]^。表皮生长因子受体（epidermal growth factor receptor, EGFR）在大多数上皮来源的肿瘤中表达增多，其过度表达与肿瘤细胞的增殖、活动、侵袭、凋亡抑制、血管生成相关。有研究^[2, 3]^表明，EGFR高表达与肿瘤治疗抵抗有一定关系，会导致肿瘤细胞对放射治疗不敏感。因此，抑制EGFR表达及其下游激活的通路可能会增加肿瘤细胞对放疗的敏感性。放疗是各期非小细胞肺癌（non-small cell lung cancer, NSCLC）患者均可采用的治疗手段，然而放化疗联合在增加对肿瘤细胞杀伤作用的同时其副反应也随之增加，所以寻找低毒性药物与放疗联合来提高肿瘤的局控率是解决的方法之一。吉非替尼（ZDl839、IRESSA、Gefitinib、易瑞沙）是一种口服的表皮生长因子受体抑制剂（epidermal growth factor receptor tyrosine kinase inhibitors, EGFR-TKIs），被用于晚期NSCLC的二线或三线治疗^[4]^。放疗可诱导EGFR自身磷酸化及其下游通路的激活，促进肿瘤细胞增殖，抑制凋亡^[5]^。因此两种治疗方法联用在理论上可能会增强NSCLC治疗效果。本实验旨在研究观察吉非替尼是否对NSCLC细胞株HCC827和H358有放疗增敏作用以及其机制。

## 1 材料与方法

### 1.1 材料

人NSCLC细胞株NCI-H358和HCC827，均购自中国医学科学院基础医学研究所基础医学细胞中心。其中NCI-H358为*EGFR*野生型，基因水平大量扩增，对吉非替尼敏感；HCC827为EGFR的E746-A750氨基酸缺失，对吉非替尼敏感。实验药品吉非替尼，由阿斯利康公司赠予，称重研磨溶于DMSO，终浓度为10 mmol/L，分装后-20 ℃保存备用。RPMI-1640培养基为吉诺生物有限公司产品，Histone一抗为Biovision公司产品，细胞核浆蛋白分离提取试剂盒为碧云天生物技术研究所产品，共聚焦显微镜为日本Olympus公司产品。

### 1.2 方法

#### 1.2.1 细胞培养

人NSCLC细胞株NCI-H358和HCC827接种于含100 mL/L加热灭火胎牛血清RPMI-1640完全培养液中，置于37 ℃、50 mL/L CO_2_、相对湿度90%的细胞培养箱中培养，细胞呈贴壁生长，取对数生长期细胞进行实验。

#### 1.2.2 细胞照射

将对数生长期细胞置于0.5 cm的补偿物上，将直线加速器机架角度为180度，以保证相同厚度的剂量建成区。应用6 MV X线按所需剂量输出剂量照射。

#### 1.2.3 克隆形成实验测定吉非替尼对H358和HCC827细胞的放疗增敏作用

取对数生长期细胞，用PBS洗2遍后，消化制成细胞悬液。用细胞计数板计数后稀释细胞到合适密度，再按不同照射剂量接种合适细胞数到60 mm直径的平皿，每个剂量设3个副孔。细胞贴壁后对照组只换液，2株细胞HCC827和H358均分别分为2组：单纯X线照射组、X线+吉非替尼组，实验组加1 µmol/L吉非替尼作用24 h后分别用0 Gy、2 Gy、4 Gy、6 Gy、8 Gy射线照射，然后置培养箱中培养10天-14天，其间可换液1次-2次。待培养到14天时，PBS漂洗后用甲醇固定20 min，0.1%结晶紫染色15 min，自然干燥后低倍显微镜下计数≥50个细胞的克隆数。以0 Gy组计算克隆形成效率（plating efficicy, PE），计算各个剂量下的细胞存活率（survival fraction, SF），PE=（0 Gy剂量下集落数/细胞接种数）×100%，SF=某一剂量照射组细胞形成的克隆数/（该组细胞种植数×PE），根据单击多靶模型SF2=exp（-αD-βD^2^）拟合曲线计算得到SF2值，重复3次。

#### 1.2.4 免疫荧光

胰酶消化细胞接种24孔板，使细胞在盖玻片上贴壁生长，两株细胞H358和HCC827均分别分为2组：单纯X线照射组、X线+吉非替尼组，实验组加1 µmol/L吉非替尼作用24 h，以4 Gy X线照射后予以PBS洗，4%多聚甲醛于放疗后不同时间固定15 min，PBS洗3遍，0.2% Triton X-100室温作用15 min，PBS洗3遍，5%山羊封闭血清室温1 h，加Ⅰ抗1:80（r-H2AX; EGFR）4 ℃过夜，PBS洗3遍，避光FITC-Ⅱ抗（1:50）37 ℃孵育50 min，PBS洗，1 µg/mL Hoechst 33342染核15 min，PBS洗，甘油封片，固定至载玻片上，避光放置，激光共聚焦显微镜观察。

#### 1.2.5 免疫印记

以4 Gy X线照射细胞，X线+吉非替尼组以1 µmol/L吉非替尼提前24 h预处理，以放疗后不同时间胰酶消化，离心收集细胞，加裂解液提取胞浆胞核蛋白，BCA法测蛋白浓度备用。制备5%浓缩胶，8%分离胶，以60 µg蛋白上样，电泳，先180 mA转膜90 min，再350 mA转膜90 min，取出EGFR膜，5%脱脂奶粉室温封闭90 min，Ⅰ抗（EGFR 1:1, 000; histone 1:500）4 ℃过夜，PBS洗，HRP-Ⅱ抗室温孵育2 h，PBS洗，ECL室温5 min，于暗室胶片曝光。

### 1.3 统计学方法

采用SPSS 11.5统计软件进行分析。3次独立实验的数据采用Mean±SD表示，实验组与对照组的组间差异比较采用*t*检验分析，*P* < 0.05为差异具有统计学意义。

## 2 结果

### 2.1 克隆形成实验拟合剂量生存曲线

克隆形成实验结果显示，X线+吉非替尼组H358细胞相比单纯X线组的生存曲线有明显差异，两个曲线之间分开较大（图 1），该图中，横坐标为放疗剂量，纵坐标为SF值取对数，如图所示，X线+吉非替尼组H358的SF2值明显小于单纯放疗组，计算出SF2值，单纯X线组SF2值为0.433，加药组SF2值为0.355，说明加药组H358细胞对放疗更敏感。而对于HCC827细胞，X线+吉非替尼组和单纯X线组的生存曲线无明显差异（图 2），计算出的SF2值，单纯放疗组为0.223，加药组为0.242，说明预先使用吉非替尼可能对HCC827细胞并无明显的放疗增敏效应。

**1:**
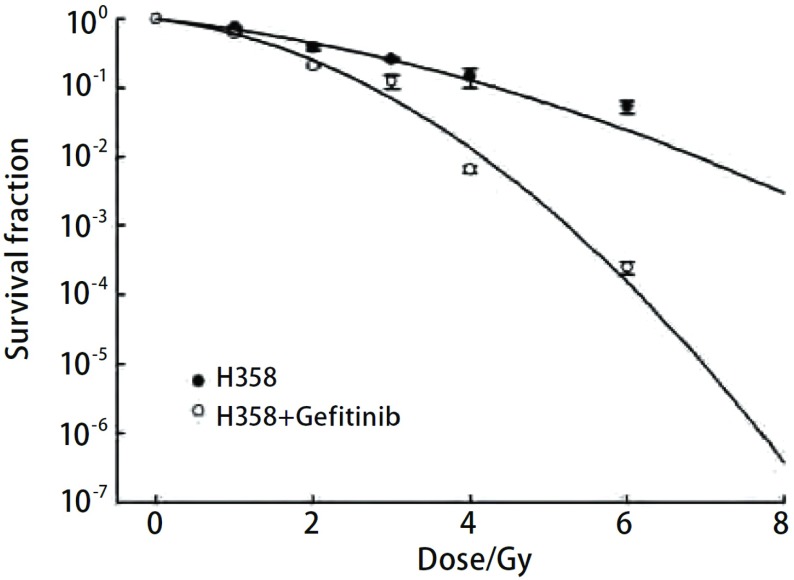
H358细胞单纯放疗组和加药组剂量生存曲线 H358 cells dose-survival curve

**2:**
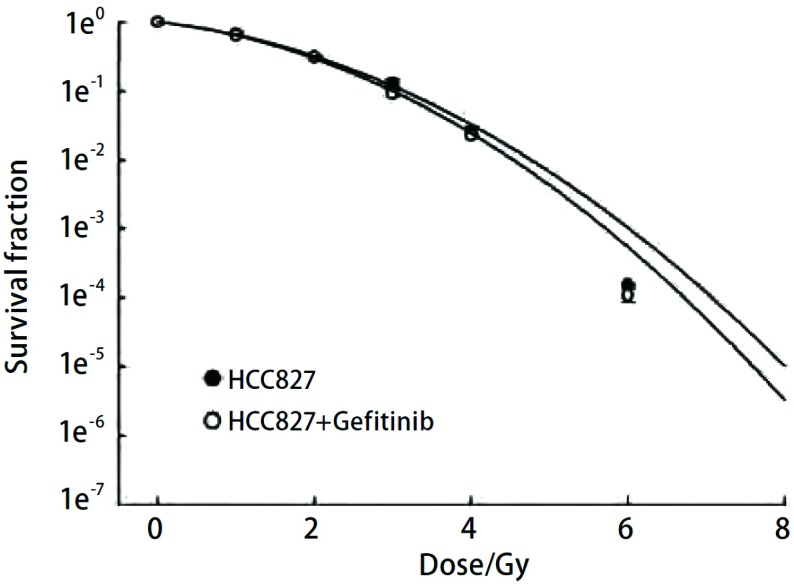
HCC827细胞单纯放疗组和加药组的剂量生存曲线 HCC827 cells dose-survival curve

### 2.2 激光共聚焦显微镜观察放疗后不同时间段细胞核内γ-H2AX表达情况

激光共聚焦显微镜观察放疗后不同时间段核内γ-H2AX焦点，对于H358细胞，图 3中蓝色底为细胞核，绿色点即为γ-H2AX焦点，结果显示，单纯X线组（图 3A）γ-H2AX焦点于放疗后30 min内开始增加，1 h γ-H2AX焦点到达高峰，随后逐渐较少，到12 h几乎没有，24 h完全没有。X线+吉非替尼组（图 3B）γ-H2AX焦点在各时间段均比单纯放疗组多，放疗后4 h仍可见较多γ-H2AX焦点，到放疗后24 h γ-H2AX焦点仍然存在。对于HCC827细胞，图 4中蓝色底为细胞核，绿色点即为γ-H2AX焦点。结果显示，单纯X线组（图 4A）和X线+吉非替尼组（图 4B）两组间的γ-H2AX焦点在放疗后各时间段表达并无明显差异。

**3:**
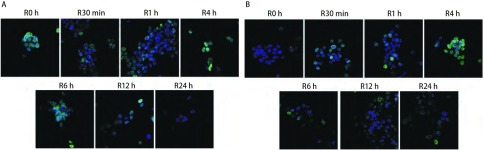
激光共聚焦显微镜观察H358细胞4 Gy放疗后不同时间段细胞核内γ-H2AX焦点的情况。A：单纯X线组；B：X线+吉非替尼组。 Immunostaining for confocal microscopy testing for nuclear γ-H2AX foci of H358 cells after 4 Gy X-ray. A: X-ray group; B: X-ray+Gefitinib group.

**4:**
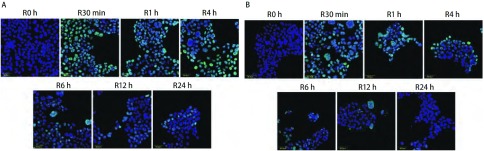
激光共聚焦显微镜观察HCC827细胞4 Gy放疗后不同时间段细胞核内γ-H2AX焦点的情况。A：单纯X线组；B：X线+吉非替尼组。 Immunostaining for confocal microscopy testing for nuclear γ-H2AX foci of HCC827 cells after 4 Gy X-ray. A: X-ray group; B: X-ray+Gefitinib group.

### 2.3 激光共聚焦显微镜观察放疗后EGFR入核情况

激光共聚焦显微镜观察放疗后EGFR入核，对于H358细胞，图 5中蓝色底为细胞核，红光则为EGFR，结果显示，单纯X线组（图 5A）EGFR在放疗后30 min开始入核，在1 h处达到高峰，X线+吉非替尼组（图 5B）EGFR在各时间段均多在细胞质中表达，无入核趋势。对于HCC827细胞，图 6中蓝色底为细胞核，红光为EGFR，结果显示，单纯X线组（图 6A）和X线+吉非替尼组（图 6B）EGFR在各时间段表达情况基本一致，即均在细胞质中表达，并无入核现象。

**5:**
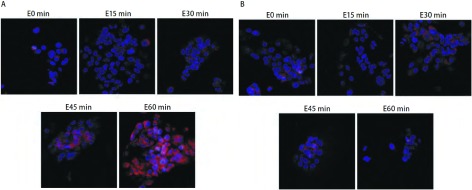
激光共聚焦显微镜观察H358细胞4 Gy放疗后不同时间段EGFR入核情况。A：单纯X线组；B：X线+吉非替尼组。 Immunostaining for confocal microscopy testing for nuclear EGFR foci of H358 cells after 4 Gy X-ray. A: X-ray group; B: X-ray+Gefitinib group.

**6:**
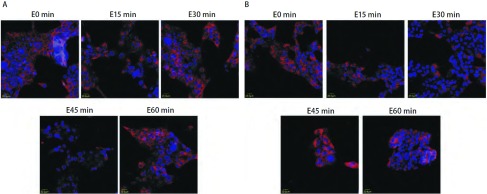
激光共聚焦显微镜观察HCC827细胞4 Gy放疗后不同时间段EGFR入核情况。A：单纯X线组；B：X线+吉非替尼组。 Immunostaining for confocal microscopy testing for nuclear EGFR foci of H358 cells after 4 Gy X-ray. A: X-ray group; B: X-ray+Gefitinib group.

### 2.4 Western blot检测放疗后胞浆胞核中EGFR表达变化情况

Western blot结果显示，对于H358细胞，图 7中单纯X线组（图 7A）EGFR在放疗后有入核情况，如图所示，从放疗后15 min开始EGFR在胞核中表达增多，到1 h处条带达最深，呈递增趋势，而胞浆中EGFR表达在各时间段则呈递减趋势，说明EGFR在放疗后从胞浆进入胞核。X线+吉非替尼组（图 7B）EGFR则均在胞浆中表达，且条带无明显差异，核内EGFR表达很少。采用凝胶成像灰度定量系统计算各条带的灰度值，并做成柱状图（图 7C），对两组数据进行配对*t*检验，差异有统计学意义（*P*=0.031），对照组的灰度值均高于实验组。这说明H358细胞在放疗后1 h内，在各个相同的时间点，X线+吉非替尼组细胞核EGFR表达均少于单纯X线组，可能是由于吉非替尼通过某种途径阻止EGFR放疗后进核。对于HCC827细胞，单纯X线组（图 8A）和X线+吉非替尼组（图 8B）两组EGFR在各时间段均在胞浆中表达，胞核中很少或不存在，且胞浆中条带并无趋势差异。采用凝胶成像灰度定量系统计算各条带的灰度值，并做成柱状图（图 8C），对两组数据进行配对*t*检验，差异无统计学意义（*P*=0.32），这说明HCC827细胞在放疗后1 h内，在各个相同的时间点，单纯X线组和X线+吉非替尼组这两组中EGFR表达并无差异，且EGFR蛋白在胞核中表达明显少于胞浆。

**7:**
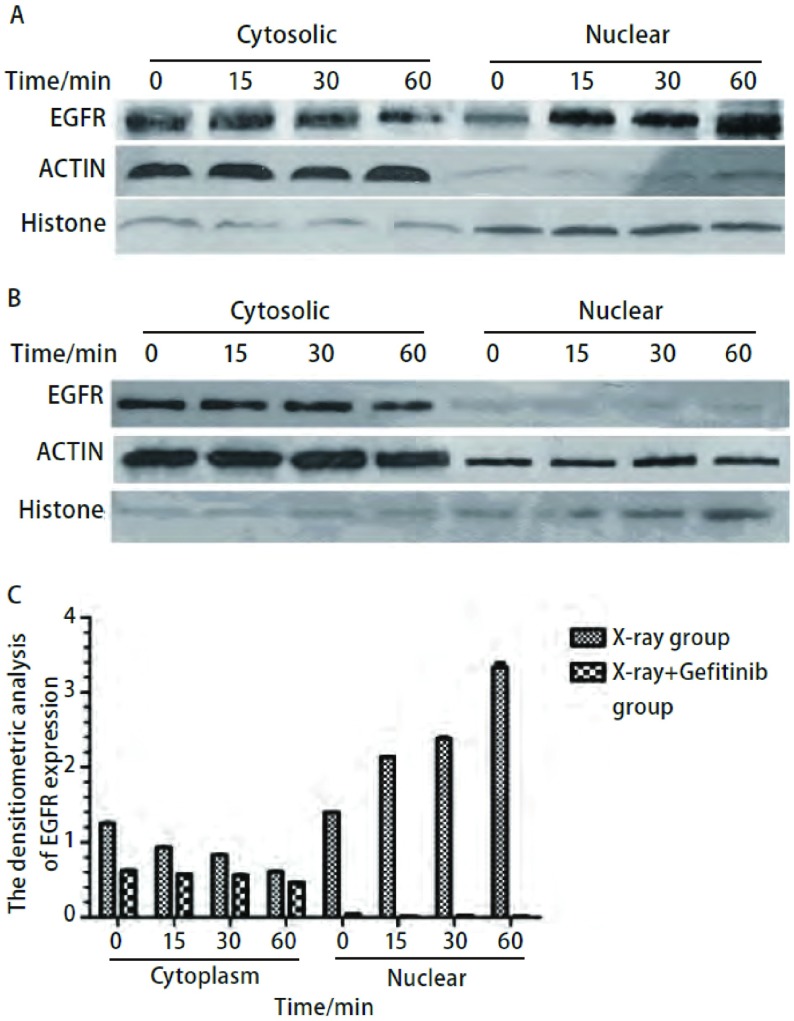
Western blot检测H358细胞4 Gy放疗后不同时间点胞浆胞核中EGFR表达变化情况。A：单纯X线组；B：X线+吉非替尼组；C：EGFR带灰度定量柱状图。 Western blot testing for cytoplasm and nuclear EGFR of H358 cells after 4 Gy X-ray. A: X-ray group; B: X-ray+Gefitinib group; C: The densitometric analysis of EGFR expression.

**8:**
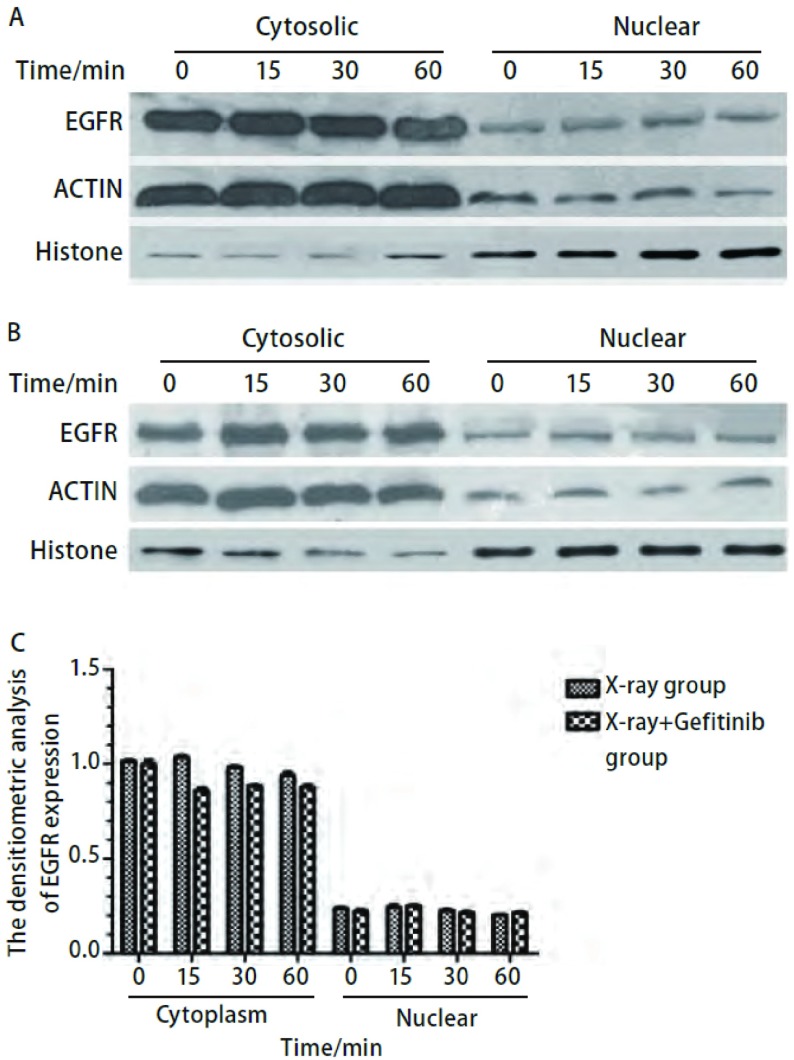
Western blot检测HCC827细胞4 Gy放疗后不同时间点胞浆胞核中EGFR表达变化情况。A：单纯X线组；B：X线+吉非替尼组；C：EGFR带灰度定量柱状图。 Western blot testing for cytoplasm and nuclear EGFR of HCC827 cells after 4 Gy X-ray. A: X-ray group; B: X-ray+Gefitinib group; C: The densitometric analysis of EGFR expression.

## 3 讨论

EGFR是跨膜糖蛋白受体Erb-B家族成员之一，属于酪氨酸激酶生长因子受体，此类受体在正常细胞和肿瘤细胞中具有一定程度的表达，与细胞增殖、分化、凋亡、血管生成等密切相关。当EGFR与相应配体（如EGF、TNF-α、Neuregulin等）结合或暴露于电离辐射、紫外线、高温、乏氧等环境中时，会与受体活化形成同源或异源二聚体形式，导致胞内酪氨酸激酶活化，促进受体自身磷酸化，激活下游转导通路如Ras/Raf/MEK/ERK、PLCv/DAG/PKC、JAK/SRC/STAT以及PBK/AKT途径，导致细胞异常增殖和分化、血管生成增加并抑制肿瘤细胞凋亡^[6]^。以放疗为主的综合治疗是局部晚期NSCLC的标准治疗方案，电离辐射可引起DNA发生各种类型的损伤，其中双链断裂（double strands break, DSB）是最致命的损伤形式，因此，抑制放疗后DSB修复是增加肿瘤放射敏感性的核心因素。大量体外研究显示，EGFR参与了DSB损伤修复的过程，能够通过多种途径来影响肿瘤细胞的放射敏感性。暴露于电离辐射，可以激活EGFR，使核外的EGFR在不依赖配体的情况下进入到细胞核内，与DSB修复的重要蛋白DNA-PK形成蛋白复合体，促进DNA-PKc磷酸化，实现DSB修复^[7-11]^。另外，EGFR也可通过激活下游转导通路，如Ras/Raf/MEK/ERK/MAPK及PI3K/PDK1/Akt（PKB）通路来调节细胞凋亡、增殖、周期循环，并影响DSB修复相关蛋白的基因转录，从而影响放疗敏感性^[12, 13]^。目前针对EGFR的靶向治疗已成为NSCLC治疗的热点。EGFR单克隆抗体C225已被单药或与化/放疗联合用于临床。有研究者^[14]^以头颈部鳞癌细胞系SCC-13Y作为对象，观察发现不论是单次照射还是分次照射，C225均可降低其存活分数，增加放疗后细胞的凋亡率，表现为放射增敏效应。还有研究^[15]^发现，C225通过抑制放疗后EGFR入核与DNA修复相关蛋白DNA-PK结合，增加了肺癌细胞株A549和乳腺癌细胞株MDA MB231的放疗敏感性。本研究旨在探讨吉非替尼是否有与C225相似的放射增敏效应及其可能的机制。

本实验选取两株NSCLC细胞株，分别是H358和HCC827。其中H358为*EGFR*野生型，在基因水平大量扩增，对吉非替尼敏感；HCC827为EGFR的E746-A750氨基酸缺失，对吉非替尼敏感。根据两株细胞的IC_50_以及吉非替尼的半衰期，选取1 µg/mL吉非替尼作为增敏浓度。

本实验根据两细胞株放疗后克隆形成情况，计算SF值绘制剂量生存曲线。可以看到H358细胞X线+吉非替尼组各个剂量的细胞存活率均低于X线组，SF2值也同样如此。说明前者放疗后发生增殖性死亡的细胞多于后者，大部分细胞因丧失了继续增殖生长的能力而不能形成克隆，即吉非替尼使H358细胞的放疗敏感性增加。而HCC827细胞实验组及对照组SF2值并无明显差异。克隆形成实验证明吉非替尼可提高细胞株H358的放疗敏感性，而对细胞株HCC827无明显放疗增敏效应。

组蛋白H2AX在DNA发生DSB时发生磷酸化，磷酸化的H2AX参与DNA损伤修复和维持基因组稳定性；由于γ-H2AX的数量与DNA损伤的数量存在一一对应的关系，因此，γ-H2AX可以作为低剂量电离辐射衡量肿瘤放疗敏感性的指示因子^[16]^。为验证吉非替尼是否有促进放疗后引起的DNA损伤的作用，我们通过共聚焦荧光显微镜观察了放疗后不同时间段细胞核内γ-H2AX的表达。结果显示，对于H358细胞，两组均可观察到放疗后细胞核内γ-H2AX的表达，且在放疗后1 h达到高峰，后逐渐减少。但X线+吉非替尼组的γ-H2AX焦点在各个时间点均明显多于单纯X线组，且持续时间更长，这说明了吉非替尼有增加放疗后DSB的效应，使得H358细胞放疗后DSB形成增多，并延迟了DNA损伤的修复，表现出放疗增敏的作用。对于HCC827细胞，两组γ-H2AX焦点的变化及持续时间并无特殊差异，说明放疗后两组DSB大致相同，吉非替尼的预先处理并未增加HCC827细胞的放射敏感性。

为确定两株细胞放疗后是否有EGFR入核作用，我们通过共聚焦荧光显微镜观察了两株细胞放疗后EGFR的表达位置变化情况。结果显示，对于H358细胞，单纯X线组EGFR在放疗后1 h内进入细胞核，并在1 h处达到高峰；而X线+吉非替尼组EGFR光点在各个时间段几乎都位于细胞质内，细胞核内很少或不存在。而对于HCC827细胞，两组的EGFR在1 h内不同时间点均在细胞质中表达，细胞核内很少或不存在，两组并无特殊差异。这说明放疗后H358细胞EGFR入核，而HCC827细胞EGFR不入核。而相关研究^[17]^也有显示，野生型*EGFR*在放疗后可入核，并与DNA修复蛋白DNA-PK结合完成放疗后DSB的修复，从而达到放疗保护的作用；而突变型*EGFR*放疗后不入核，不能与DNA-PK结合，从而表现出对放疗的敏感。也有研究^[18]^发现，如果把突变型*EGFR*导入对放疗耐受的野生型NSCLC细胞，且阻断野生型*EGFR*的放疗保护作用，拥有突变型*EGFR*的该细胞不仅表现出其原有的放疗保护作用丧失，而且产生了明显的放射增敏效应。

为进一步验证两株细胞放疗后EGFR入核情况，我们用Western blot检测了放疗后两株细胞胞质、胞核中EGFR的表达。结果显示，对于H358细胞，单纯X线组在放疗后1 h内胞浆EGFR表达随时间减少，胞核EGFR表达随时间增多，在1 h达到高峰，而X线+吉非替尼组EGFR均在细胞质表达，且无变化趋势，细胞核内很少或几乎没有EGFR表达；对于HCC827细胞，两组EGFR在胞质和胞核中的表达在各个时间点并无趋势差异，且组间也无特殊差异，这与共聚焦荧光显微镜所观察到的结果相同。由于EGFR可通过放疗激活进行下游信号通路的传导，其中野生型*EGFR*可直接在放疗后入核，与DNA-PKcs结合进行DSB修复起到放疗保护的作用，因此我们可以推测，吉非替尼可能是通过抑制放疗后EGFR入核与DNA修复蛋白DNA-PK结合来增加放疗效应。

有研究^[19]^选取多株不同类型NSCLC细胞株进行了体外实验，结果显示，表达突变型*EGFR*的细胞对放疗表现出敏感性，出现放疗后DNA修复受阻；而相反，表达野生型*EGFR*的多株细胞则出现放疗后有效的DNA修复，并推论出突变型*EGFR*可能通过阻碍放疗后引起的DSB修复而达到放疗增敏作用。还有研究结果^[9]^显示，对于表达野生型*EGFR*的细胞，在受到电离辐射后，可以入核与DSB修复的关键蛋白DNA-PK结合，并可通过免疫共沉淀检测出EGFR-DNA-PKcs或EGFR-Ku80的复合体，而这一结果在表达突变型*EGFR*的细胞中并未发现。最近有研究^[20, 21]^指出，吉非替尼的放疗增敏作用可能与其抑制了放疗后ATM的表达和活性有关，而这一作用在别的小分子EGFR酪氨酸激酶抑制剂如而厄洛替尼中却并未发现，且吉非替尼的放疗增敏效果受细胞株中COX-2的表达影响，过表达COX-2的细胞容易对吉非替尼产生耐药从而使其不能发挥放疗增敏作用。

然而，吉非替尼是否还通过其它途径影响H358细胞和HCC827细胞的放疗敏感性；是否阻止EGFR入核后与DNA修复相关蛋白DNA-PKCs的结合来达到增敏作用；抑制DNA-PKcs的活性是否会对核内EGFR的表达及放疗敏感性产生影响；体内实验与体外实验是否能得出相同的结论；吉非替尼与放疗联用是否有积极的临床价值，以上问题还需要进一步的体外及体内实验来验证。

本课题来源于：

1.吴阶平医学基金会基金《基于EGFR不同基因型的NSCLC个体化放疗机制初探》（No.320.6720.10013; 2011.01-2012.12）。

2.国家自然科学基金《EGFR备选非小细胞肺癌放疗增敏靶点的机制研究》(No.30801351; 2009.1-2011.12）。
